# Prognostic role of systemic inflammation indexes in metastatic urothelial carcinoma treated with immunotherapy

**DOI:** 10.2144/fsoa-2023-0049

**Published:** 2023-06-24

**Authors:** Michele Dionese, Umberto Basso, Francesco Pierantoni, Eleonora Lai, Nicolò Cavasin, Elisa Erbetta, Salim Jubran, Giorgio Bonomi, Davide Bimbatti, Marco Maruzzo

**Affiliations:** 1 Oncology Unit 1, Istituto Oncologico Veneto, IOV – IRCCS, Padova, 35128, Italy; 2 Oncology Unit 3, Istituto Oncologico Veneto, IOV – IRCCS, Padova, 35128, Italy; 3 Department of Surgery, Oncology, & Gastroenterology, University of Padova, Padova, 35128, Italy

**Keywords:** bladder carcinoma, immune-related adverse events, immunotherapy, neutrophil-to-lymphocyte ratio, urothelial carcinoma

## Abstract

**Aims:**

Inflammation indexes had been associated with overall survival (OS) and immune-related adverse events (irAEs) in patients treated with immune checkpoint inhibitors (ICIs).

**Materials & methods:**

in 72 patients treated with ICIs for metastatic urothelial carcinoma (mUC) we evaluate differences in OS, response rate and toxicities, according to baseline inflammation indexes values.

**Results:**

neutrophil-to-lymphocite ratio (NLR) <3 was associated to longer progression-free survival (PFS; 4.9 vs 3.1 months) and OS (15.7 vs 7.6 months); monocyte-to-lymphocite ratio (MLR) <0.4 was associated to longer PFS (4.6 vs 2.8 months). Overall response rate (ORR), disease control rate (DCR) were higher in these patients. Patients with an irAE had longer PFS and OS.

**Conclusion:**

baseline inflammatory indexes are prognostic for mUC patients treated with ICIs.

## Plain language summary

Between 2019 and 2021, 72 patients received immunotherapy for a metastatic urothelial carcinoma at our institution. We detected markers of inflammation from a common blood test (neutrophils, lymphocytes, platelets and monocytes). We analyzed the role of these markers in our patients. Patients with a lower ratio of these biomarkers at baseline had a significantly longer time to disease progression. They also has a longer survival and higher chance of disease reduction. Moreover, patients with a specific ratio (NLR) increasing more than 0.5, after the first cycle of therapy, had a worse prognosis. It is necessary to evaluate the role of these markers in a trial with new patients enrolled in a prospective way. In fact, the role of these marker in predicting survival and adverse events could be very important for patients' management.

## Tweetable abstract

Systemic inflammation indexes (NLR, MLR) are associated to PFS, OS, ORR and DCR in metastatic urotelial carcinoma, treated with immunotherapy.

## Graphical abstract

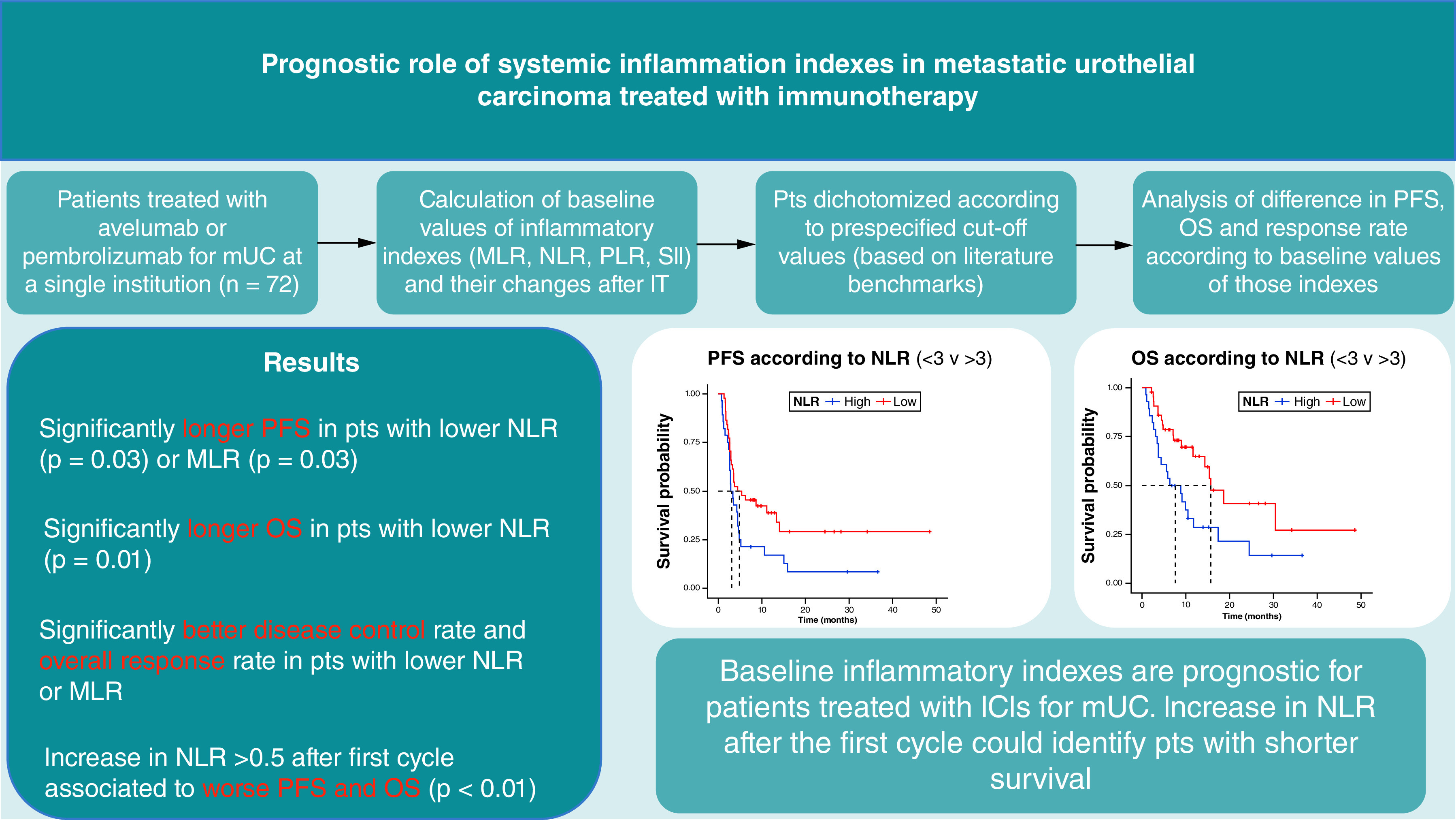

Urothelial carcinoma is one of the most prevalent types of cancer in the world, with a long-term prognosis for patients with advanced disease that is still dismal [1]. In recent years, immune checkpoint inhibitors (ICIs) became the standard treatment after first line chemotherapy [2,3]. Pembrolizumab had shown in a phase III trial to prolong overall survival (OS) compared with chemotherapy as second line treatment after progression to chemotherapy [4,5]. More recently, for patients that completed a first line platinum-based chemotherapy without radiological progression at the last CT scan, a maintenance treatment with avelumab demonstrated to prolong median OS compared with observation only [6,7]. Other ICIS such as nivolumab and durvalumab have been studied for second line indication but they are not available in every country.

Over the years, many clinical factors and circulating biomarkers have demonstrated prognostic value in UC patients treated with systemic therapies. Metastases in specific sites (liver, bone, or other visceral organs), low hemoglobin levels, elevated markers of systemic inflammation and a compromised performance status have been associated with a worse prognosis in patients treated with both traditional chemotherapy and immunotherapy [8–11].

Recently, certain systemic inflammation markers calculated using peripheral blood count values (such as the NLR or the platelet-to-lymphocyte ratio, PLR) were shown to be prognostic for patients with urothelial carcinoma and other solid malignancies [12–17] Moreover, for patients treated with ICIs for metastatic neoplasms, variation of baseline blood markers after the first cycles of therapy could be associated to progression free survival (PFS) and overall survival (OS) [18,19] In addition, for patients affected by non-small cell lung cancer, had been shown to identify patients at higher risk to develop immune-related adverse events (irAEs) [20].

Our aim was therefore to study the prognostic role and the possible correlation with development of irAEs of baseline systemic inflammation markers in a real-world cohort of patients treated with ICIs for mUC.

## Materials & methods

### Design & objective of the study

We designed a retrospective observational study in order to evaluate the prognostic role of inflammatory biomarkers in patients treated with avelumab or pembrolizumab for mUC in a real-world setting.

The primary objective was to find a possible correlation between baseline inflammatory indexes and overall survival. Secondary objectives were to evaluate the association between baseline inflammatory indexes and progression-free survival, radiological responses, the development of immune-related toxicities, and to investigate the prognostic role of changes in inflammatory biomarkers after the first immunotherapy cycle.

### Patients

We enrolled all consecutive patients at our institution who received pembrolizumab or avelumab for metastatic urothelial carcinoma (mUC) after platinum-based chemotherapy, administered either in the neo-adjuvant, adjuvant or in the first-line metastatic setting. Drugs were administered as per label, in second-line (pembrolizumab) or in first-line maintenance (avelumab) for metastatic disease. Clinical data were extracted from electronic patient records.

Inclusion criteria were a histological diagnosis of UC of the bladder or upper urinary tract, the presence of metastases, and at least one course of treatment with pembrolizumab or avelumab for advanced disease.

At the time of their first visit to our institution, all patients consented to the use of their clinical data for scientific purposes.

We collected baseline clinical data (in particular blood count values at baseline and after the first cycle), tumor characteristics, details on immunotherapy treatment,immunotherapy-related toxicity (type and grade, date of development, interruption of treatment, use of corticosteroids or second-level immunosuppressive agents), and date of death or last follow-up.

We calculated inflammatory indexes for each patient both at baseline and after the first immunotherapy cycle. Inflammatory indexes were: neutrophil-to-lymphocyte ratio (NLR: neutrophils/lymphocytes), platelet-to-lymphocyte ratio (PLR: platelets/lymphocytes), monocyte-to-lymphocyte ratio (MLR: monocytes/lymphocytes), and systemic immune-inflammation index (SII: neutrophils x platelets/lymphocytes). Patients were dichotomized according to pre-specified cut-off values for NLR (≥3 vs <3), PLR (≥150 vs <150), SII (≥1375 vs <1375), and MLR (≥0.4 vs <0.4); cut-off values were based on literature benchmarks [10,12–15,21].

We also calculated the Bellmunt prognostic score for each patient (one point each for ECOG performance status >0, hepatic metastases, and hemoglobin level <10 g/dl) [9]. Adverse events were graded according to the Common Terminology Criteria for Adverse Events (CTCAE) v5.0 [22]; radiological response was defined using Response Evaluation Criteria in Solid Tumors (RECIST) v1.1 criteria [23].

### Treatment

Pembrolizumab or avelumab was administered in accordance with the approved label as per clinical practice. Radiological evaluations, blood tests, and other diagnostic tests were performed in line with the established standard of care.

### Statistical analysis

The Kaplan–Meier method was used to calculate progression-free survival (PFS) from the date of treatment initiation to the date of disease progression or death from any cause (whichever occurred first); PFS was censored at the last patient follow-up visit without progression. Overall survival was measured from the date of drug initiation until the date of death from any cause or censored at the last follow-up.

Key metrics were summarized by means of descriptive statistics. Categorical variables were compared with the Chi-squared test or Fisher's exact test. Significant differences in ordinal variables were checked using the Mann-Whitney test. The correlation between inflammatory markers was calculated using the Spearman method.

PFS and OS were compared using the log rank test and Cox's proportional hazards method. We performed univariate and multivariate analyses to determine the association between baseline characteristics and PFS and OS; covariates with a p-value less than 0.1 that showed any association with the oncologic outcome in the univariate analysis were included in the multivariate analysis. The assumptions of proportional hazards were verified before applying Cox models to the analyses using the Shoenfeld residuals method.

Results were classified as statistically significant if their p-values were <0.05. All statistical analyses were conducted using the “R” software v4.0.5 and the “survival” package v2.44–1.1.

## Results

### Patient characteristics

A total of 72 patients were treated for mUC with pembrolizumab (49; 68.1%) or avelumab (23; 31.9%) at our institution between January 2019 and December 2021. Patients' demographic data and principal clinical characteristics are summarized in Table 1.

**Table 1.** Patients characteristics (n = 72).

Characteristics	Patients, n (%)
Sex: Male Female	56 (77.8%) 16 (22.2%)
Age (years) Median (range) >70 years (%)	68.4 (51.3–88.6) 30 (41.7%)
Surgery for localized disease Yes No	50 (69.4%) 22 (30.6%)
Location of primary tumor Bladder UTUC	55 (76.4%) 17 (23.4%)
Stage at diagnosis M0 M1	49 (68%) 23 (32%)
Histology Pure UC UC with hist. variants	60 (83.3%) 12 (16.7%)
Metastatic sites Visceral (any site) Bone Liver Lung Lymph nodes Others	47 (65.3%) 26 (36.1%) 17 (23.6%) 30 (41.7%) 54 (75%) 18 (25%)
Basal ECOG PS 0 1 2	45 (62.5%) 23 (31.9%) 4 (5.6%)
Blood tests Hb (range) Hb <10 g/dl (%)	11.5 (8.7–15.4) 13 (18.1%)
Prior CT Cisplatin + gemcitabine Carboplatin + gemcitabine	52 (72.2%) 20 (27.8%)
Bellmunt score 0 1 2 3	37 (51.4%) 18 (25.0%) 14 (19.4%) 3 (4.2%)

FGFR: Fibroblast growth factor receptor; Hb: Hemoglobin; MLR: Monocyte-to-lymphocyte ratio; NLR: Neutrophil-to-lymphocyte ratio; PLR: Platelet-to-lymphocyte ratio; PS: Performance status (ECOG); SII: Systemic immune-inflammation index; UTUC: Upper tract urothelial carcinoma.

The median age of the cohort was 68.4 years, with approximately more than a third (30; 41%) of the patients being older than 70 years. Most of the patients (76.4%) had urothelial carcinoma developing in the bladder. The majority of patients had localized disease at diagnosis (68%); hence, most of the patients underwent surgery with curative intent. A high proportion of patients (47; 76%) had visceral metastases at the start of immunotherapy; 52 patients were treated with cisplatin-based chemotherapy, while the remaining patients received a carboplatin-based regimen.

### PFS, OS & response

After a median follow-up of 13.9 months, 53 patients (73.6%) progressed and 39 (54.2%) died.

The median PFS of patients treated with pembrolizumab was 3.5 months (95% CI: 2.8–10.6 months), while the median OS was 10.5 months (95% CI: 6.3–18.6). The median PFS of patients treated with avelumab maintenance was 5.2 months (95% CI: 2.8-NR months), while the median OS was not reached at the time of follow-up (95% CI: 9.83-NR).

For patients treated with pembrolizumab, the best response to treatment was complete response in 2 patients (4.1%), partial response in 12 patients (24.5%), stable disease in 5 patients (10.2%), and progression in 30 patients (61.2%); therefore, disease control was achieved in 19 patients (disease control rate, DCR: 38.8%) and the objective response rate (ORR, defined as the proportion of patients with a radiological response to treatment) was 28.6%. For patients treated with avelumab maintenance, the best response to treatment was partial response in 6 patients (26.1%), stable disease in 7 patients (30.4%), and progression in 10 patients (43.5%); the disease control rate and overall response rate were 56.5% and 26.1%, respectively.

### Baseline blood count & systemic inflammatory indexes

Table 2 presents baseline blood counts and calculated inflammation indexes at first immunotherapy cycle. In the whole population, 28 patients (38.9%) had an NLR >3, 40 patients (55.5%) PLR >150, 15 patients (20.8%) SII >1375 and 25 patients (34.7%) MLR >0.4.

**Table 2.** Baseline blood values at the start of immunotherapy.

	Median	Mean	Range
Neutrophil (n × 10^∧^9/l)	4.15	4.97	1.32–23.3
Lymphocytes (n × 10^∧^9/l)	1.61	1.72	0.66–3.59
Platelets (n × 10^∧^9/l)	251	288	106–1084
Monocytes (n × 10^∧^9/l)	0.54	0.59	0.16–1.52
NLR	2.57	3.29	0.67–14.84
PLR	156.9	190.8	49.7–690.5
SII	658	1122	109–16088
MLR	0.33	0.38	0.10–1.13

MLR: Monocyte-to-lymphocyte ratio; NLR: Neutrophil-to-lymphocyte ratio; PLR: Platelet-to-lymphocyte ratio; SII: Systemic immune-inflammation index.

### Analysis of baseline prognostic factors

Tables 3 & 4 depict the results of a univariate analysis of prognostic factors for PFS and OS.

**Table 3.** Association between baseline inflammatory indexes and PFS and OS in univariate analysis.

Characteristic	n	PFS (m)	HR (95% CI)	p-value	OS (m)	HR (95% CI)	p-value
NLR >3 <3	28 44	3.1 4.9	0.56 (0.32–0.96)	**0.03**	7.6 15.7	0.45 (0.24–0.84)	**0.01**
PLR >150 <150	40 32	3.3 5.8	0.67 (0.38–1.16)	0.2	9.1 15.7	0.57 (0.29–1.11)	0.15
SII >1375 <1375	15 57	2.8 3.7	0.71 (0.38–1.34)	0.3	6.3 15.3	0.62 (0.31–1.26)	0.2
MLR >0.4 <0.4	25 47	2.8 4.6	0.56 (0.32–0.97)	**0.03**	8.8 15.3	0.55 (0.29–1.04)	0.06

Bold values indicate statistically significant data (p less than 0.05).

m: Months; MLR: Monocyte-to-lymphocyte ratio; NLR: Neutrophil-to-lymphocyte ratio; PLR: Platelet-to-lymphocyte ratio; SII: Systemic immune-inflammation index.

**Table 4.** Analysis of additional characteristics associated with PFS and OS in the univariate analysis.

Characteristic	N	PFS (m)	HR (95% CI)	p-value	OS (m)	HR (95% CI)	p-value
Histology Transitional Other/rare	60 12	4.4 2.6	1.65 (0.82–3.32)	0.2	14.3 4.7	1.92 (0.9–4.06)	0.10
Surgery on T No Yes	20 52	2.8 5.1	0.37 (0.21–0.66)	**<0.01**	7.1 17.3	0.39 (0.19–0.77)	**<0.01**
Age, years <70 >70	42 30	3.5 4.1	0.96 (0.55–1.66)	0.9	10.5 15.3	1.01 (0.53–1.92)	1
First-line tx Carboplatin-based Cisplatin-based	20 52	3.0 4.3	0.61 (0.34–1.09)	0.10	11.7 14.3	0.64 (0.32–1.29)	0.2
Status at diagnosis M0 M1	49 23	6.2 2.8	3.16 (1.78–5.62)	**<0.01**	17.3 6.3	2.85 (1.45–5.63)	**<0.01**
Visceral mets No Yes	24 48	4.6 3.1	1.53 (0.86–2.74)	0.3	17.3 11.6	1.61 (0.80–3.21)	0.2
Bone mets No Yes	46 26	7.4 2.8	2.71 (1.55–4.75)	**<0.01**	24.5 4.7	4.5 (2.30–8.80)	**<0.01**
Liver mets No Yes	55 17	4.4 2.9	1.18 (0.63–2.21)	0.6	11.7 7.1	1.42 (0.71–2.87)	0.3
ECOG PS 0 1–2	45 27	5.3 2.5	2.41 (1.39–4.16)	**<0.01**	15.7 4.7	2.95 (1.55–5.63)	**<0.01**
Hb basal >10 <10	59 13	4.4 2.7	1.48 (0.74–2.95)	0.3	15.3 6.3	1.75 (0.82–3.74)	0.1
Bellmunt 0 1 2 3	37 18 14 3	5.3 2.7 2.8 1.6	1.80 (0.94–3.45) 1.76 (0.86–3.6) 5.88 (1.72–20.1)	**0.01**	15.7 5.3 6.3 2.5	2.52 (1.18–5.38) 2.35 (0.98–5.61) 7.41 (2.08–26.3)	**<0.01**

Hb: Hemoglobin; m: Months; Mets: Metastasis; PS: Performance status (ECOG); T: Primary tumor; Tx: Treatment.

**Figure 1.**
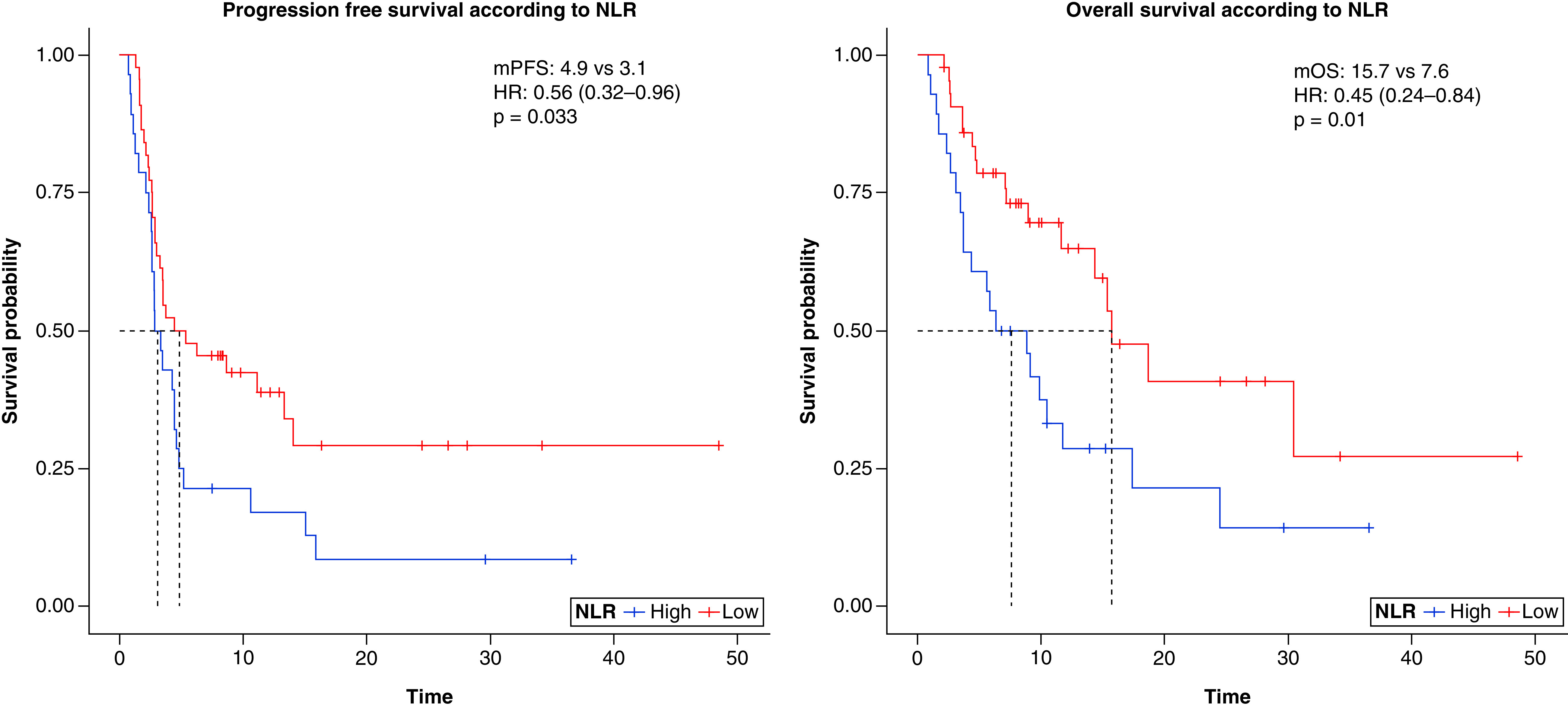
Progression-free survival (left) and overall survival (right) in patients with neutrophil-to-lymphocite ratio >3 or <3. HR: Hazard ratio; NLR: Neutrophil-to-lymphocite ratio; OS: Overall survival; PFS: Progression-free survival.

Patients with an NLR inferior to 3 had longer PFS (4.9 vs 3.1 months; HR 0.56, 95% CI: 0.32–0.96) and longer OS (15.7 vs 7.6 months; HR 0.45, 95% CI: 0.24–0.84) than patients with an NLR >3 (Table 3); both differences were statistically significant (p of 0.03 and 0.01, respectively). Figure 1 reports the survival curves as a function of NLR values.

Of the 49 patients treated with pembrolizumab, those with an NLR >3 had shorter OS (5.8 vs 15.7 months, p = 0.02) and shorter PFS (2.8 vs 3.6 months, p = 0.08), although the difference was not statistically significant. Of the 23 patients treated with avelumab, those with an NLR >3 had shorter PFS (3.30 vs 5.8 months) and OS (9.8 vs not reached); however, the differences were not statistically significant (p of 0.3 and 0.5) probably due to the low numbers in this cohort.

Patients with an MLR inferior to 0.4 had a statistically significant improvement in PFS (4.6 vs 2.8 months; HR 0.56, 95% CI: 0.32–0.97; p = 0.03). In our cohort, patients with an MLR <0.4 also had a longer overall survival (15.3 vs 8.8 months; HR 0.55, 95% CI: 0.29–1.04), with borderline statistical significance (p = 0.06).

A PLR greater than 150 and an SII greater than 1375 were not found to be significantly associated with a poorer PFS and OS (Table 3).

Additional factors associated with longer PFS in the univariate analysis were localized disease at diagnosis, previous surgical treatment on the primary tumor, the absence of bone metastasis, a low ECOG PS (0), and a low Bellmunt prognostic score (Table 4). The same prognostic factors were also associated with a longer OS (Table 4).

Only the presence of metastasis at the time of first diagnosis and a high Bellmunt score were associated with a poorer PFS in the multivariate analysis. The same two factors, along with the presence of bone metastasis and an NLR >3, were independently associated with a poorer OS (Table 5).

**Table 5.** Multivariate analysis for PFS and OS.

Characteristic	HR for PFS (95% CI)	p-value	HR for OS (95% CI)	p-value
Stage at diagnosis (M1 vs M0)	2.68 (1.45–4.93)	**<0.01**	2.29 (1.11–4.75)	**0.02**
Bone M (Yes vs No)	2.00 (0.99–4.07)	0.06	3.81 (1.76–8.25)	**<0.01**
Bellmunt score 0 1 2 3	– 1.12 (0.51–2.49) 1.13 (0.51–2.51) 5.27 (1.46–19.1)	– 0.76 0.77 **0.01**	– 1.14 (0.49–2.65) 1.29 (0.50–3.30) 6.19 (1.67–22.9)	0.76 0.59 **<0.01**
NLR (<3 vs >3)	0.72 (0.41–1.29)	0.27	0.49 (0.25–0.95)	**0.03**

Bold values are statistically significant.

M: Metastasis; NLR: Neutrophil-to-lymphocyte ratio.

### Radiological response & inflammatory indexes

Both ORR and DCR were significantly higher in patients with a low NLR compared with those with an NLR >3 (ORR: 36.3% vs 14.3%, p = 0.04; DCR: 54.5% vs 28.6%, p = 0.03, respectively).

Patients with a lower MLR had a significantly higher ORR and DCR than patients with baseline MLR >0.4 (ORR: 36.2% vs 12%, p = 0.03; DCR: 53.2% vs 28%, p = 0.04, respectively). Patients with lower PLR or SII values had a better ORR and DCR (PLR: ORR 31.8% vs 21.4% and DCR 50% vs 35.7%, respectively; SII: ORR 29.8% vs 20% and DCR 47.4% vs 33.3%, respectively); however, the differences were not statistically significant (Table 6).

**Table 6.** Radiological response as a function of PLR, MLR, and SII.

	Pts (n)	Progressive disease (40 pts)	Stable disease (12 pts)	Partial response (18 pts)	Complete response (2 pts)
NLR >3 <3	28 44	20 (71.6%) 20 (45.5%)	4 (14.3%) 8 (18.2%)	4 (14.3%) 14 (31.8%)	0 (0%) 2 (4.5%)
PLR >150 <150	40 32	26 (65%) 14 (43.8%)	5 (12.5%) 7 (21.9%)	9 (22.5%) 9 (28.1%)	0 (0%) 2 (6.2%)
SII >1375 <1375	15 57	10 (66.7%) 30 (52.7%)	2 (13.3%) 10 (17.5%)	3 (20%) 15 (26.3%)	0 (0%) 2 (3.5%)
MLR >0.4 <0.4	25 47	18 (72%) 22 (46.8%)	4 (16%) 8 (17.1%)	3 (12%) 15 (31.9%)	0 (0%) 2 (4.2%)

MLR: Monocyte-to-lymphocyte ratio; NLR: Neutrophil-to-lymphocyte ratio; PLR: Platelet-to-lymphocyte ratio; SII: Systemic immune-inflammation index.

### Variations in blood tests & inflammatory indexes & correlation with response

We examined variations in the values of complete blood count and inflammatory indexes after the first dose of immunotherapy for 69 eligible patients whose results were available.

There were no significant changes in blood count values or systemic inflammation indexes after the first cycle of immunotherapy (ΔNLR p = 0.38, ΔPLR p = 0.25, ΔSII p = 0.98).

We then evaluated NLR values for each patient, considering a decrease/increase greater than a minimum cut-off of 0.5 proved to detect a significant variation.

An increase in NLR greater than 0.5 was found in 21 patients; a decrease in NLR greater than -0.5 was found in 18 patients; no variations in NLR greater than 0.5 were found in the remaining 30 patients.

An increase in NLR >0.5 after the first cycle was associated with worse PFS (2.8 vs 5.3 months, p < 0.01) and OS (5.8 vs 18.6 months, p < 0.01) in the entire population.

### Immune-related adverse events

Of the whole population, 29 patients (40.3%) developed immune-related adverse events of various grades. The most common adverse events reported were skin toxicities (pruritus and rash, 16 patients, 22.2%), followed by diarrhea (8 patients, 11.1%), endocrinological and metabolic disorders (8 patients hypothyroidism, 2 patients hyperglycemia), and elevation of liver enzymes (6 patients, 8.3%). The majority of patients (27, 37.5% of the total) experienced G1–G2 toxicities. Only 2 patients (2.8%) in our cohort reported G3–G4 toxicities (a G3 diarrhea and a G3 nephritis).

The mean time to the onset of an iRAE was 106 days (range: 14–577 days). Eighteen patients developed a single-type AE, while the others patients developed more than one type of AE. Due to AE, 9 patients (12.5%) temporarily discontinued therapy, which was then resumed, while 2 patients (2.8%) discontinued treatment permanently. Twelve patients in total (16.7%) were administered systemic corticosteroids. The results are summarized in Table 7.

**Table 7.** Immune-related adverse events reported in our cohort.

Type of iRAE	Grade 1–2	Grade 3–4	Management of the AE		
			Temporary interruption	Systemic steroids	Permanent discontinuation
Skin AEs	16 (22.2%)	0 (0%)	3 (4.2%)	6 (8.3%)	1 (1.4%)
Diarrhea	8 (11.1%)	1 (1.4%)	4 (5.6%)	4 (5.6%)	0 (0%)
Hypothyroidism	8 (11.1%)	0 (0%)	0 (0%)	0 (0%)	0 (0%)
Hyperglycemia	2 (2.8%)	0 (0%)	0 (0%)	0 (0%)	0 (0%)
Liver enzyme elevation	6 (8.3%)	0 (0%)	0 (0%)	0 (0%)	0 (0%)
Pneumonia	1 (1.4%)	0 (0%)	0 (0%)	0 (0%)	0 (0%)
Asthenia	2 (2.8%)	0 (0%)	0 (0%)	0 (0%)	0 (0%)
Arthralgia	2 (2.8%)	0 (0%)	0 (0%)	1 (1.4%)	0 (0%)
Renal AEs	1 (1.4%)	1 (1.4%)	2 (2.8%)	2 (2.8%)	1 (1.4%)
Total patients	28 (38.9%)	2 (2.8%)	9 (12.5%)	12 (16.7%)	2 (2.8%)

AE: Adverse event.

Grade 1–2 toxicities were more common in patients with a low NLR (45.5% vs 28.6%) or a low MLR (44.7% vs 28.0%); however, the association was not statistically significant (p = 0.21 and p = 0.23, respectively). All the inflammatory index values were above the cut-off for the two patients who developed G3–G4 irAEs.

Patients who developed an irAE of any grade had longer PFS (15.1 vs 2.6 months; HR 0.19, 95% CI: 0.10–0.38, p < 0.01) and OS (30.4 vs 6.3 months; HR 0.25, 95% CI: 0.12–0.52, p < 0.01) than those who did not.

## Discussion

Immunotherapy has dramatically altered the therapeutic landscape of metastatic urothelial carcinoma. Pembrolizumab was found to be superior to second-line chemotherapy in terms of radiological response and OS in patients who progressed after platinum-based treatment [4,5]. Avelumab was the first drug to prolong PFS and OS as a maintenance treatment in patients with a radiological response or stable disease following first-line platinum-base treatment [6,7]. Despite the evident benefits in terms of prolonged survival and an improved quality of life, a significant proportion of patients treated with immunotherapy experience rapid disease progression and do not benefit from the treatment.

Our population represents a homogeneous, real-world cohort of patients treated with on-label pembrolizumab or avelumab in clinical practice. The clinical and demographic characteristics of patients enrolled in our studies were comparable to those enrolled in clinical trials. Survival outcomes and radiological response were consistent with data from randomized trials and other real-world experiences [4–7].

In recent years, along with other established prognostic factors, several authors have studied the role of systemic inflammation indexes as prognostic biomarkers (either as single parameters or within combined prognostic scores) for patients treated with ICIs in many solid tumors [12,14,18,20,24]. Composite indexes may be able to simultaneously evaluate the imbalance between systemic inflammation (neutrophils, monocytes, and platelets) and the patient's immune-competence (lymphocytes).

Regarding the treatment of mUC, Fornarini *et al.* analyzed a real-world cohort of more than 250 patients treated in Italy with atezolizumab in the phase IIIb SAUL trial and found a significant association between survival outcomes (PFS and OS), NLR (cut-off: 3, 3.65, and 5), and SII (cut-off: 884 and 1375) [12]. On analyzing all patients enrolled in the trial, they confirmed a prognostic role for NLR (HR for OS: 1.66 and 2.17 for NLR cut-off 3 and 5, respectively) and SII (HR for OS: 2.16 and 2.19 for SII cut-off 884 and 1375, respectively); consequently, they were able to create a novel prognostic score for OS [25].

Sonpavde *et al.* confirmed the role of NLR using patient-level data from phase I/II trials investigating ICI for mUC. They developed a five factor prognostic score (LDH, liver metastasis, NLR, ECOG PS, and platelets) and validated the score in two different trial populations [24]. They confirmed a robust association between NLR (considered a continuous variable with no cut-off value) and OS and incorporated the parameter into a multi-factor prognostic score (HR for logNLR: 2.30) [24].

Kadono *et al.* found that MLR, PLR, NLR (considered both continuously and with a cut-off of 2.9), and SIRI (systemic inflammation response index) were all associated with OS [13].

To the best of our knowledge, there are no data in the literature on the association between baseline inflammatory indexes and oncological outcomes in patients treated with avelumab maintenance for UC.

In our population, patients with a high NLR, MLR, SII, or PLR had shorter PFS and OS; the difference, however, was only statistically significant for NLR (PFS and OS) and MLR (PFS).

Regarding the response rate, patients with higher values of NLR and MLR had a significantly worse DCR and ORR. It is interesting to note that the two patients who achieved complete response to treatment had low values for all the inflammatory indexes considered in the study.

Many authors have also investigated the role of changes in blood count values after the initiation of immunotherapy, including for other tumors. In genitourinary oncology, in a large multicenter study involving 422 patients treated with nivolumab for metastatic renal cell carcinoma, authors reported a significant increase in both blood count values and inflammatory indexes after initiation of treatment and found that an increase in NLR greater than 0.5 after the first cycles was associated with worse PFS and OS [18]. Regarding UC, an increase in NLR was also associated with shorter OS in the above-cited research by Kadono and colleagues [13].

We did not find a significant variation in blood count or inflammatory indexes after the first cycle of immunotherapy. However, variations may develop even after months of treatment [18] and, therefore, these may not have been documented in our study due to the follow-up time. We were able to corroborate the data, in that an increase in NLR during treatment with ICIs or an increase in NLR >3 after the first cycle of therapy results in a poorer prognosis for patients [13,18].

The issue of the optimal cut-off values for each parameter remains open to debate. Our analyses relied on commonly used cut-off values rather than calculating the optimal cut-off value for each parameter using receiver operating characteristic curves. We view this as a strength, as the data are highly comparable to previously published data.

Toxicities were manageable in our study; only 2 patients were required to permanently discontinue treatment due to adverse events. The safety profile of our population is consistent with the published literature.

As reported for several other solid malignancies, there was an association between the development of irAEs and improved outcomes in our cohort [20,26]. This evidence currently lacks a definitive biological explanation. However, we can hypothesize that the time required for the development of adverse events could result in a selection bias in favor of patients with better outcomes. We were unable to find baseline risk factors for the development of irAEs in our cohort. Low-grade toxicities seemed to be more common in patients with a low NLR and MLR (patients with a better prognosis and treated for a longer period of time), but the results were not statistically significant. In contrast, the 2 two high-grade toxicities reported in our study occurred in patients with high values in all systemic inflammation indexes.

Classical prognostic factors for UC, such as performance status, the Bellmunt score, and the presence of visceral, liver, or bone metastases, were confirmed in our cohort [8,9,11]. However, not all of the results were statistically significant, probably due to the relatively small number of patients in our population.

This study's main limitation is its retrospective design, with potential selection bias. Patients were restaged at different time points according to local practice, and this may have artificially prolonged PFS for some patients compared with the more stringent radiological schedules typically required in clinical trials. Moreover, no independent second review of CT scans was conducted. Although the sample size was not small in absolute terms, this prevented us from performing a subgroup analysis of the prognostic value of inflammatory markers by analyzing the two treatments separately. Blood tests were conducted in different laboratories. Finally, the PD-L1 status of these patients was not investigated [27].

## Conclusion

Our data confirm that treatment with avelumab and pembrolizumab in a homogeneous, real-world population is safe and achieves comparable oncological outcomes to those reported in clinical trials. However, patients' long-term outcomes remain unsatisfactory.

Despite the limitations concerning the study's retrospective nature and small population, we confirmed that NLR and MLR are prognostic factors for survival and response in ICI-treated patients. The toxicity profile was manageable. Inflammatory indexes appeared capable of identifying patients with a higher risk of developing high-grade toxicities, but this result needs to be validated in larger and prospective trials.

More data will be generated from ongoing multicenter trials exploring the prognostic and predictive role of inflammation indexes in urothelial cancer patients treated with immunotherapy; these results may improve patient selection in clinical practice.

**Summary points**

- We investigated the prognostic role of baseline systemic inflammation markers in a real-world cohort of patients treated with immunotherapy for metastatic urothelial carcinoma.
- This was a retrospective, observational study that included 72 consecutive patients treated, between 2019 and 2021, with pembrolizumab or avelumab at a single institution.
- Baseline neutrophil-to-lymphocite ratio (NLR) <3 was associated to significantly longer progression-free survival (PFS; 4.9 vs 3.1 months, p = 0.03), longer overall survival (OS; 15.7 vs 7.6 months, p = 0.01) and higher overall response rate (ORR) and disease control rate (DCR; 36.3% vs 14.3% and 54.5% vs 28.6% respectively).
- Baseline monocyte-to-lymphocite ratio (MLR) <0.4 was associated to significantly longer PFS (4.6 vs 2.8 months, p = 0.03) and higher ORR and DCR (36.2% vs 12% and 50% vs 35.7% respectively).
- Patients with NLR increasing more than 0.5 after the first cycle of immunotherapy had significantly shorter PFS and OS (2.8 vs 5.3 months and 5.8 vs 18.6 months respectively, p values <0.01).
- Low grade toxicities were more common in patients with low NLR or low MLR, but differences were not statistically significant.
- Comprehensively, outcomes and safety of pembrolizumab and avelumab in a real-world population were consistent with data from scientific publications.
- Systemic inflammation indexes deserve to be evaluated in a prospective study due to the possible association of baseline values with survival and adverse events.
